# *Legionella* suppresses the host unfolded protein response via multiple mechanisms

**DOI:** 10.1038/ncomms8887

**Published:** 2015-07-29

**Authors:** Sean Treacy-Abarca, Shaeri Mukherjee

**Affiliations:** 1 Department of Microbiology and Immunology, University of California, San Francisco, 513 Parnassus Avenue, San Francisco, California 94143-0552, USA; 2 Department of Microbiology and Immunology, George Williams Hooper Foundation, 513 Parnassus Avenue, Box 0552, Rm HSW 1522, San Francisco, California 94143-0552, USA

## Abstract

The intracellular pathogen, *Legionella pneumophila*, secretes ∼300 effector proteins to modulate the host environment. Given the intimate interaction between *L. pneumophila* and the endoplasmic reticulum, we investigated the role of the host unfolded protein response (UPR) during *L. pneumophila* infection. Interestingly, we show that the host identifies *L. pneumophila* infection as a form of endoplasmic reticulum stress and the sensor pATF6 is processed to generate pATF6(N), a transcriptional activator of downstream UPR genes. However, *L. pneumophila* is able to suppress the UPR and block the translation of prototypical UPR genes, BiP and CHOP. Furthermore, biochemical studies reveal that *L. pneumophila* uses two effectors (Lgt1 and Lgt2) to inhibit the splicing of XBP1u mRNA to spliced XBP1 (XBP1s), an UPR response regulator. Thus, we demonstrate that *L. pneumophila* is able to inhibit the UPR by multiple mechanisms including blocking XBP1u splicing and causing translational repression. This observation highlights the utility of *L. pneumophila* as a powerful tool for studying a critical protein homeostasis regulator.

## Abstract (web summary)

The bacterium *Legionella pneumophila*, a causative agent of severe pneumonia, replicates inside an endoplasmic reticulum-like organelle in the host cells. Here, Treacy-Abarca and Mukherjee show that the pathogen dampens the host’s unfolded protein response (UPR) pathway by multiple mechanisms.

## Graphical abstract

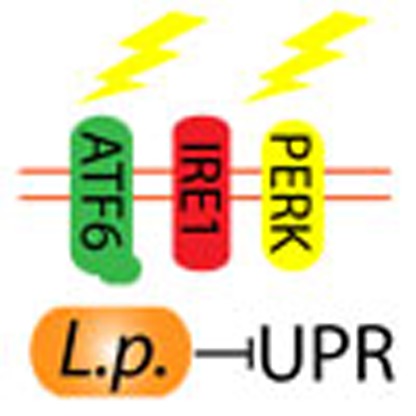

L*egionella pneumophila* is a gram negative intracellular pathogen that is responsible for a severe pneumonia known as Legionnaires’ disease. The ability to survive and replicate within eukaryotic cells is an essential hallmark of *L. pneumophila* virulence. Thus, understanding *L. pneumophila*’s ability to modulate the cellular environment is key in understanding its ability to cause disease. After phagocytosis into alveolar macrophages, *L. pneumophila* avoids phago-lysosome fusion by modifying the plasma membrane-derived vacuole into an endoplasmic reticulum (ER)-like replicative vacuole known as the *Legionella*-containing vacuole (LCV). *L. pneumophila* uses the Type IV (Dot/Icm) secretion apparatus to inject bacterial proteins into the host cell. Translocation of bacterial effectors is required for the establishment and maintenance of the LCV. *L. pneumophila* actively utilizes an arsenal of ∼300 secreted bacterial effectors to modulate cellular pathways during infection to provide the pathogen optimal conditions for replication. The secreted effectors enable *L. pneumophila* to manipulate key cellular pathways such as protein trafficking, autophagy, immune response and host chromatin remodelling among others^1^,^2^.

The unfolded protein response (UPR) pathway is an important cytoprotective pathway that is highly conserved in eukaryotes^3^. It is primarily responsible for maintaining protein homeostasis by responding to and controlling misfolded proteins. The UPR is activated by three main sensors of ER stress—inositol requiring enzyme-1 (IRE1), protein kinase RNA-like ER kinase (PERK) and activating transcription factor 6 (ATF6; ref. 4). The downstream consequences of an activated UPR to various forms of ER stress are well characterized and include global translation inhibition^5^, upregulation of specific ER stress proteins, and during extreme ER stress, activation of pro-apoptotic pathways^6^. Key hallmarks of the UPR include the splicing of XBP1u mRNA^6^, upregulation of ER chaperone protein BiP^7^ and the induction of pro-apoptotic factor CHOP^8^,^9^,^10^. Despite the critical role for the UPR in cellular homeostasis, whether and how *L. pneumophila* modulates this response is poorly understood. Given the close interaction between *L. pneumophila* and the ER, we found this question intriguing.

Control of the UPR is essential for a subset of viral pathogens which lack endogenous machinery to produce proteins. Previous studies have shown that viral pathogens such as the human cytomegalovirus are able to inhibit the detrimental conditions induced during cellular ER stress and simultaneously take advantage of the optimal conditions created by an activated UPR^11^. More specifically, human cytomegalovirus down regulates IRE1 to repress the UPR via the viral protein M50 which directly interacts with the N-terminal region of IRE1. Modulation of IRE1 via M50 has been proposed as a novel mechanism by which human cytomegalovirus restores protein homeostasis despite massive demand for viral glycoproteins during infections^12^. Previous studies have also identified links between the UPR and bacterial pathogens^13^,^14^,^15^,^16^,^17^,^18^,^19^,^20^,^21^. The UPR can be critical in detecting pathogens and many bacterial pathogens have been shown to activate the UPR. This is not surprising given that the UPR is indeed a stress response pathway that feeds into the inflammatory response^22^,^23^,^24^,^25^. However, bacterial inhibition of the UPR has only recently been described in *Simkania negevensis*, but the exact mechanism and function remain unknown^26^. It was hypothesized that inhibition of the UPR is dependent on ER contact sites that the pathogen shares with the host.

Here, we show the first evidence of bacterial inhibition of the UPR by an intracellular bacterial pathogen that is independent of direct ER contact sites. Interestingly, *L. pneumophila* is able to modulate two distinct arms of the UPR using separate mechanisms. This observation is consistent with previous studies that show that *L. pneumophila* targets the same cellular pathway with multiple effectors^27^. We show that X-box binding protein 1 (XBP1)u mRNA splicing is inhibited by the *L. pneumophila* effectors Lgt1 and Lgt2. Additionally, *L. pneumophila* is able to block the expression of key UPR response elements, BiP and CHOP, whose regulation is multifaceted, but partly controlled by the ATF6 branch of the UPR. Interestingly, the PERK pathway of the UPR appears to be largely unaffected by *L. pneumophila*. Taken together, our data highlights a previously undiscovered important role of bacterial inhibition of the UPR and opens a new avenue to study a central tenet of eukaryotic cell biology.

## Results

### *Legionella* suppresses the translation of UPR target proteins

**Figure 1:**
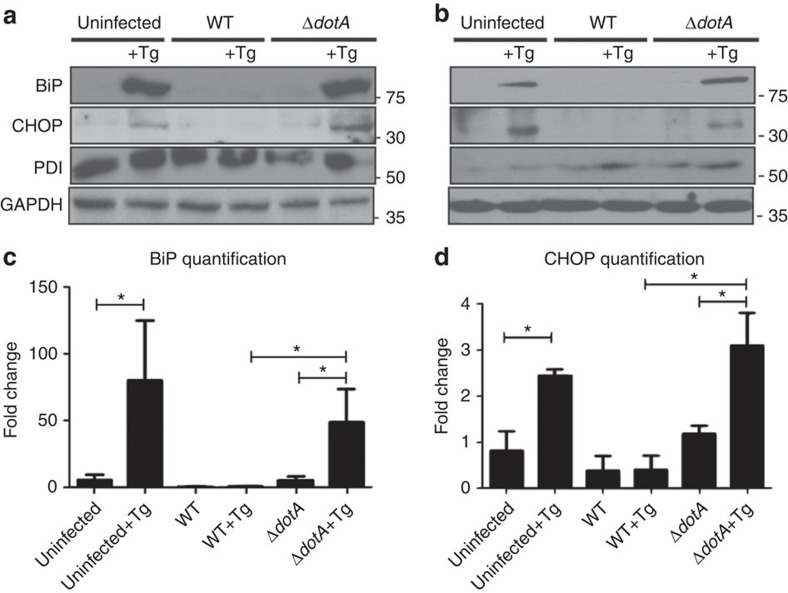
Expression of two critical components of the UPR, BiP and CHOP are blocked by *L. pneumophila* (**a**) Caspase 1/11 (−/−) bone marrow derived macrophages or (**b**) HEK-293 FCγ cells-expressing Fc gamma (HEK-293 FCγ) were either left uninfected or infected with WT or *ΔdotA* strains of *L. pneumophila* at an MOI of 150. Cells were then left untreated or treated with thapsigargin (Tg; 1 μg μl^−1^) for 6 h. BiP, CHOP and PDI protein expression were monitored via immunoblot. GAPDH was used as a loading control. Full blots are provided for Fig. 1a,b in Supplementary Fig. 5. (**c**,**d**) BiP and CHOP levels were quantified respectively from three biological replicates using the same experimental conditions in HEK-293 FCγ cells. Data are depicted as the fold change normalized to uninfected levels. Data represent three independent experiments. Values in all graphs are means±s.e.m. *P<0.05; Student’s *t*-test.

Previous studies have shown that during induction of ER stress, several genes implicated in the UPR are upregulated. Given that *L. pneumophila* infections require recruitment of ER-derived vesicles to the LCV, yet infection does not perturb the ER morphology, we investigated whether *L. pneumophila* was able to suppress the UPR during infection. As a positive control, we induced ER stress chemically using thapsigargin. Thapsigargin is a well characterized, non-competitive inhibitor of the sarco-ER Ca^2+^ ATPase and a potent inducer of ER stress^28^. Caspase 1/11 (−/−) bone marrow derived macrophages (to rule out pyroptosis) and HEK-293 cells stably expressing Fcγ receptor (to allow for opsonization of bacteria and facilitate bacterial uptake) were infected with either wild type (WT) or *ΔdotA L. pneumophila* strains and subsequently treated with thapsigargin. Interestingly, we did not observe any appreciable upregulation of either BiP or CHOP in cells that were infected with *L. pneumophila* and treated with thapsigargin (Fig. 1a–d, Supplementary Fig. 1a). In contrast, uninfected cells treated with thapsigargin showed the prototypical robust upregulation of both BiP and CHOP expression. Furthermore, an isogenic *L. pneumophila* strain, *ΔdotA*, which lacks a functional type IV secretion system, was unable to suppress the upregulation of BiP or CHOP in the presence of thapsigargin, indicating that this suppression is dependent on secreted effectors. Similar results were also obtained in macrophage-like RAW 264.7 cells using thapsigargin as well as two additional ER stress inducing compounds, Tunicamycin and dithiothreitol (Supplementary Fig. 1b). To further understand whether binding immunoglobulin protein (Bip) and C/EBP homologous protein (CHOP) expression were downregulated in a specific manner or as a result of global loss of UPR control during infection, we analysed the level of protein disulfide isomerase (PDI). PDI is another chaperone that is normally upregulated by the UPR^29^. Interestingly, we did not observe a difference in PDI expression between uninfected and *L. pneumophila*-infected cells, suggesting that *L. pneumophila* is able to specifically modulate key components of the UPR pathway (Fig. 1a,b).

### *Legionella* induces UPR genes BiP and CHOP transcriptionally

**Figure 2:**
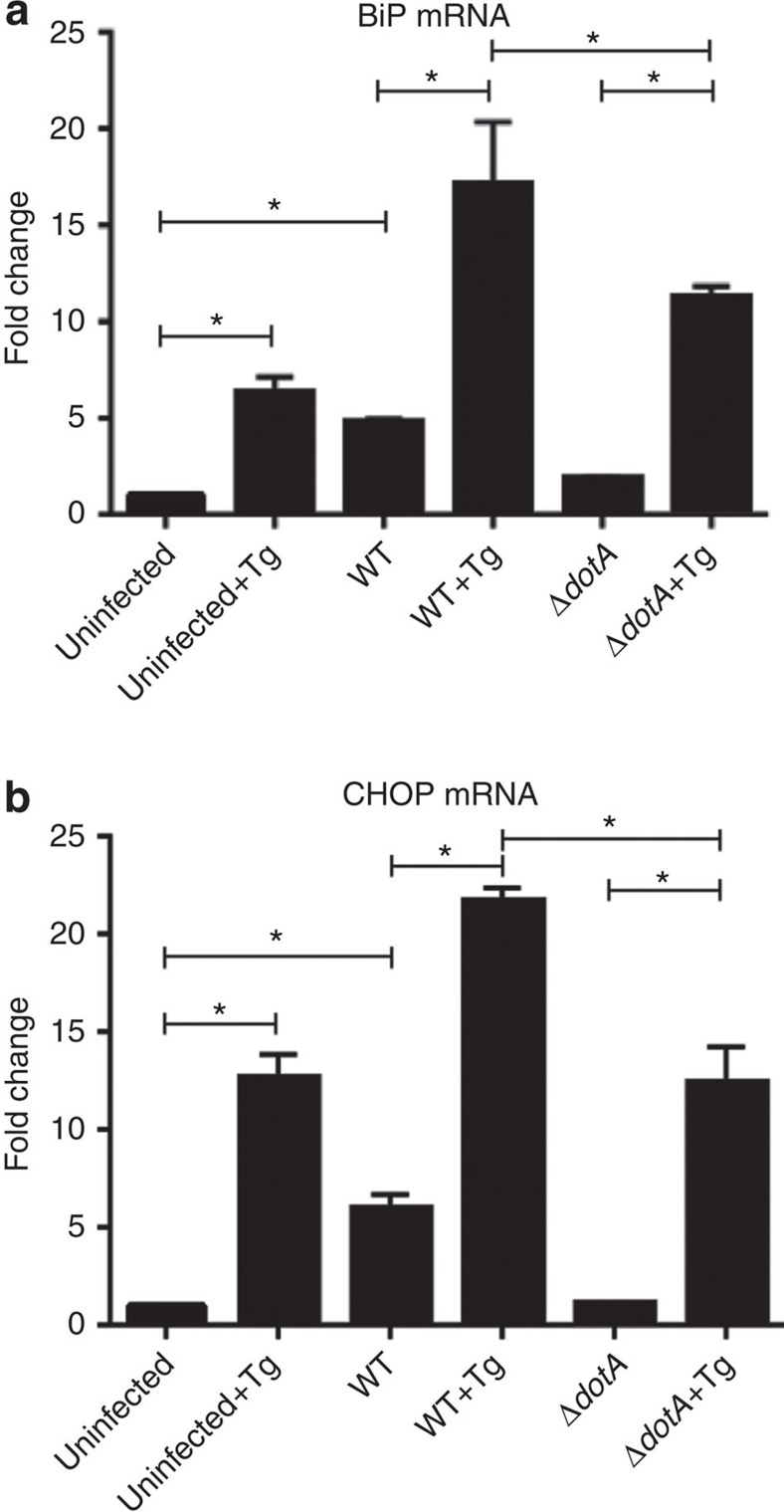
BiP and CHOP expression are not blocked transcriptionally. (**a**,**b**) Raw 264.7 cells were infected at an MOI of 150 with either WT *L. pneumophila, ΔdotA L. pneumophila*, or were left uninfected. Cells were then either untreated or treated with thapsigargin (Tg; 1 μg μl^−1^) for 6 h and mRNA was harvested and qRT–PCR was performed to assay abundance of BiP and CHOP mRNA transcripts. GAPDH was used for the endogenous normalization of gene expression. In both cases two biological replicates were used each containing three technical replicates. Values in all graphs are means±s.e.m. *P<0.05; Student’s *t*-test.

To determine if the regulation of BiP and CHOP protein accumulation by *L. pneumophila* occurs at the level of transcription or translation, we investigated BiP and CHOP mRNA abundance via quantitative reverse transcription PCR (qRT–PCR). RAW267.4 cells were treated for 6 h with thapsigargin in the presence or absence of *L. pneumophila* infection. Interestingly, WT *L. pneumophila* infection alone induced the upregulation of both BiP and CHOP mRNA levels, suggesting that host cells perceive *L. pneumophila* infection as a form of ER stress and upregulates the expression of UPR regulators (Fig. 2a,b). Furthermore, we observed that *L. pneumophila*-infected cells treated with thapsigargin did not show any defect in the transcriptional upregulation of BiP and CHOP mRNA. The induction of BiP and CHOP mRNA in *L. pneumophila*-infected cells treated with thapsigargin was significantly higher than cells treated with thapsigargin alone. Cells infected with the *ΔdotA* strain showed an induction of BiP and CHOP mRNA after thapsigargin treatment, similar to uninfected cells. Similar results were obtained when using HEK-293 Fcγ cells (Supplementary Fig. 2a,b). Additionally, we observed that both protein and mRNA levels of the Golgi protein p115 are not affected by thapsigargin treatment or infection with *L. pneumophila*, suggesting that translational regulation of BiP/CHOP is a UPR-specific phenomenon (Supplementary Fig. 2c,d). Given that the induction of BiP and CHOP is prevented at the protein level and occurred specifically in the presence of WT *L. pneumophila*, we aimed to delineate whether this was a result of the host being unable to mount the UPR as a result of being stressed in the presence of a pathogen or if this observation was linked directly to the activity of a secreted bacterial effector. Thus, we tested whether *L. pneumophila*-infected host cells were capable of mounting a response to ER stress stimulators after clearance of the *L. pneumophila* infection.

### UPR suppression by *L. pneumophila* is an effector driven process

**Figure 3:**
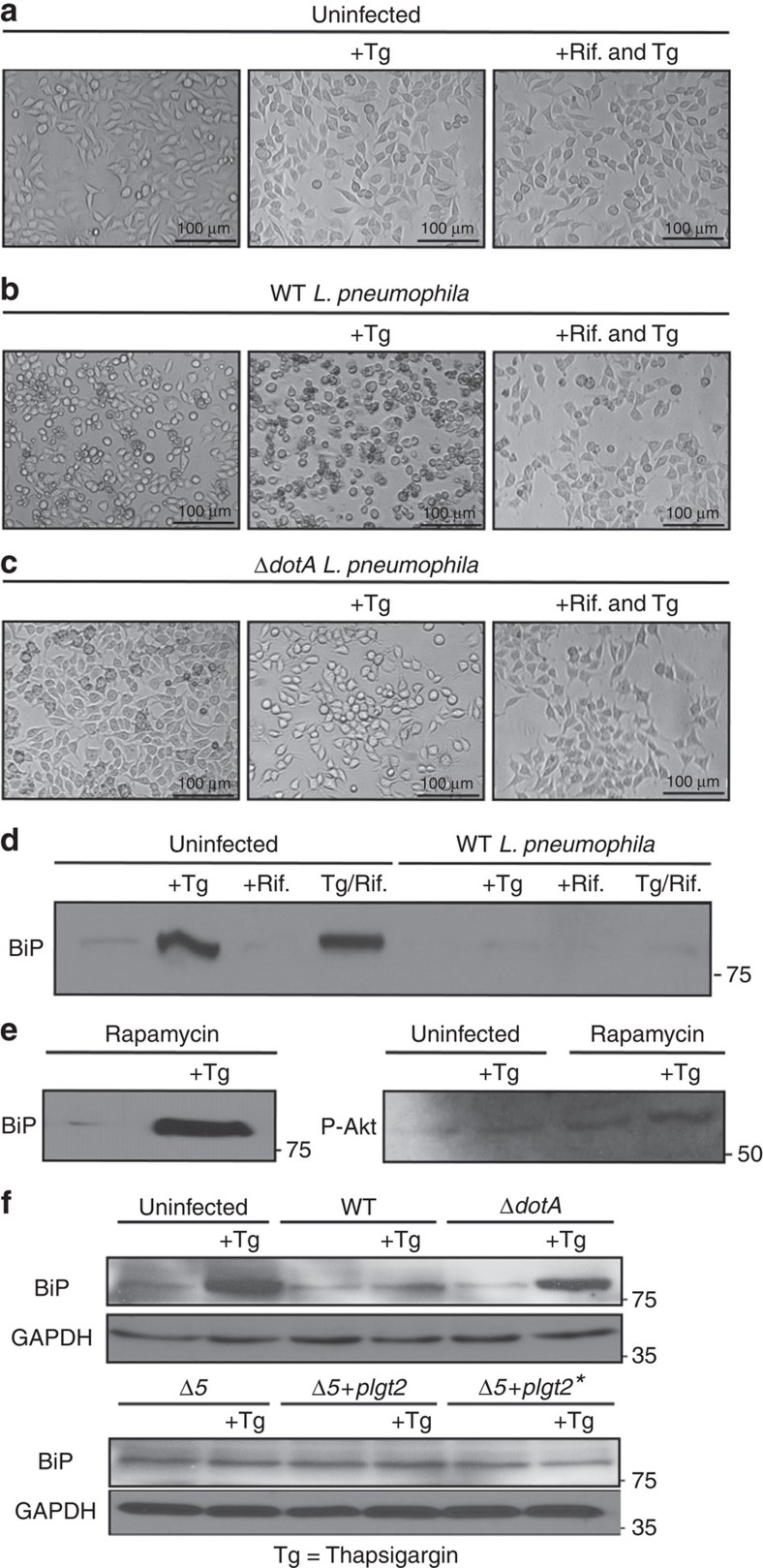
Suppression of ER stress is an effector driven process that is independent of the mTOR pathway. (**a**–**c**) HEK-293 FCγ cells were either left uninfected or infected with WT *L. pneumophila* or *ΔdotA L. pneumophila* at an MOI of 150. Cells were then either left untreated or treated with thapsigargin (Tg; 1 μg μl^−1^) or treated with a combination of Tg and the antibiotic Rifampicin (Rif.; 2 μg μl^−1^) for 6 h. After 6 h phase contrast images were taken. (**d**) HEK-293 FCγ cells were infected at an MOI of 150 with WT *L. pneumophila*, or left uninfected. Cells were then treated with Rif. alone (2 μg μl^−1^), Rif. with Tg (1 μg μl^−1^), Tg (1 μg μl^−1^) alone or left untreated for 6 h. BiP protein levels were monitored via immunoblot. (**e**) HEK-293 FCγ cells were untreated or treated with rapamycin (10 nM) or rapamycin with Tg (1 μg μl^−1^) for 6 h. BiP protein levels were then monitored via immunoblot and levels of phosphorylated Akt at serine 473 (P-Akt) were monitored from these lysates via immunoblot. (**f**) RAW 264.7 cells were infected with WT *L. pneumophila, dotA*, or Δ*5* strains of *L. pneumophila* at an MOI of 150. BiP protein expression was monitored via immunoblot. GAPDH was used as a loading control. Full blots for Fig. 3d–f are provided in Supplementary Fig. 5.

The antibiotic Rifampicin has been shown to rapidly and effectively clear *L. pneumophila* infections in both cell culture and *in vivo* models^30^. We treated *L. pneumophila*-infected cells with Rifampicin after 1 h post infection with the hypothesis that treatment with Rifampicin would clear infection, but leave behind any secreted bacterial effectors that were translocated in that time period by the Dot/Icm Type IV secretion system. As expected, cells treated with Rifampicin looked healthier compared with cells with infection alone (Fig. 3a–c). Additionally, we conducted a replicative vacuole assay to confirm that Rifampicin treatment indeed cleared *L. pneumophila* infection (Supplementary Fig. 3a,b). Strikingly, despite the treatment with Rifampicin, and the cells being healthy, BiP and CHOP protein levels were not upregulated suggesting that bacterial effectors secreted by *L. pneumophila* during the first hour post infection were able to mediate UPR suppression and that this block did not require the presence of live *L. pneumophila* (Fig. 3d).

Previous studies showed that infection with WT *L. pneumophila* (but not the *ΔdotA* strain), resulted in ubiquitination of positive regulators of mTOR kinase and led to diminished mTOR activity^31^. This resulted in a global repression of protein translation during *L. pneumophila* infection. The mTORC subunit of the mTOR1 complex is the target of the drug Rapamycin. Rapamycin inhibits mTOR by associating with its intracellular receptor FKBP12. Thus, we used Rapamycin to test whether block of BiP and CHOP protein expression was a result of inhibition of the mTOR pathway. To ensure mTOR was being inhibited we monitored the phosphorylation of Akt at serine 473. mTOR/S6K1 inhibition via Rapamycin triggers a negative feedback mechanism, resulting in the activation of Akt by phosphorylation at serine 473 via IGF-1R/PI3K (ref. 32). Interestingly, cells treated with Rapamycin were able to upregulate the expression of BiP in response to thapsigargin, suggesting that the block of the UPR by *L. pneumophila* was independent of the downregulation of the host mTOR pathway (Fig. 3e). This finding engendered our interest to identify *L. pneumophila* effectors that would directly play a role in inhibiting the UPR.

Previous studies demonstrated that five secreted *L. pneumophila* effectors (Lgt1, Lgt2, Lgt3, SidI and SidL) can use uridine diphosphate (UDP)-glucose as a substrate and modify a conserved serine residue (Ser-53) in the host elongation factor 1A (eEF1A) with a glucosyl moiety^33^. This leads to an inhibition of protein synthesis during *L. pneumophila* infection^34^. Interestingly, although a *Δ5* strain lacking these five bacterial effectors showed a defect in blocking protein translation, the strain showed no growth defects either in amoeba or macrophages^34^. One of these five effectors, Lgt2, was shown to be sufficient to rescue the block in protein translation in the *Δ5 L. pneumophila* mutant strain^34^. Given that we observed an inhibition of BiP/CHOP protein translation during UPR induction, we investigated whether these five bacterial effectors had any role in the translational block of UPR genes. To test this hypothesis, we infected cells with Δ5 mutant strain of *L. pneumophila* and assayed the accumulation of BiP during thapsigargin treatment. Despite lacking these five bacterial effectors, the Δ5 mutant strain retained the ability to block the upregulation of BiP (Fig. 3f) suggesting that additional *L. pneumophila* effectors are responsible for the block of BiP/CHOP translation during infection.

To gain an understanding of *L. pneumophila*’s impact on specific arms of the UPR, we investigated each of the three well-characterized sensors of the UPR (IRE1, ATF6 and PERK) during *L. pneumophila* infection.

### *Legionella* inhibits IRE1 signalling by blocking XBP1u mRNA splicing

**Figure 4:**
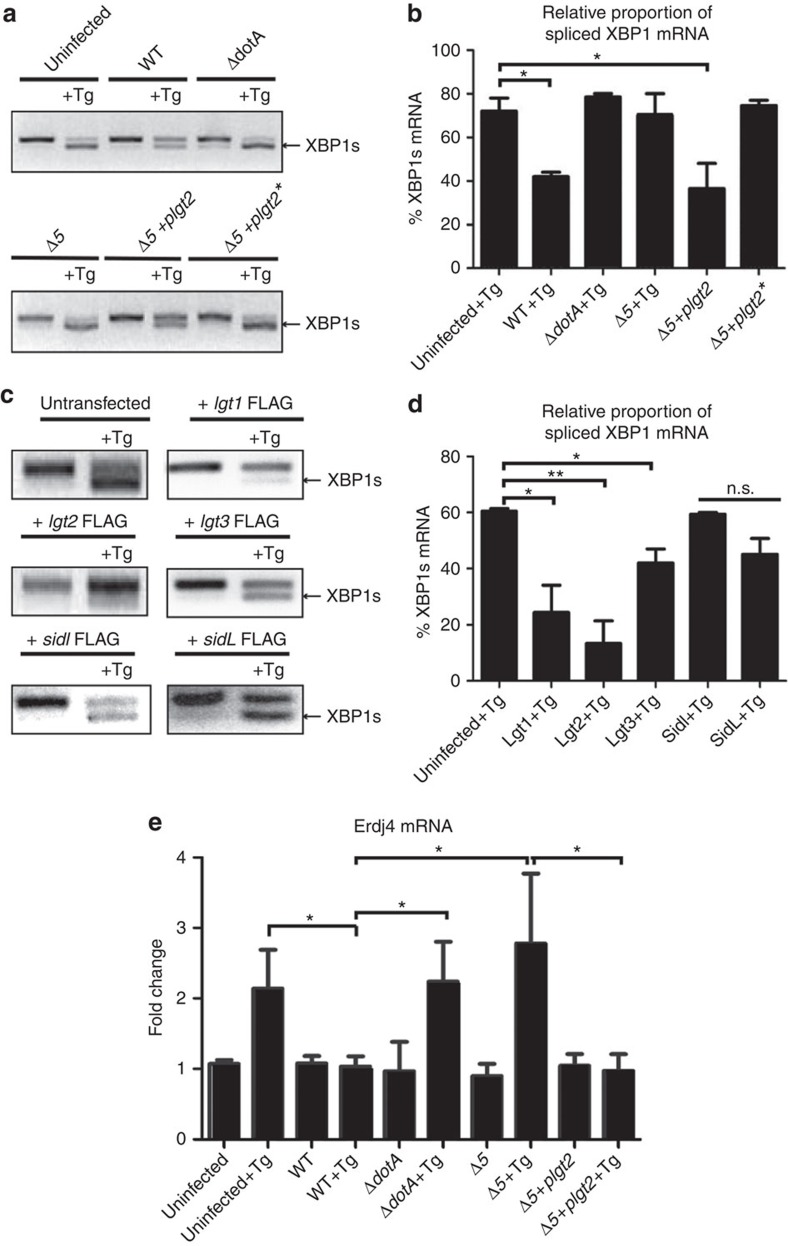
The *L. pneumophila*effector Lgt2 is necessary and sufficient to block XBP1 mRNA splicing. (**a**) RAW 264.7 cells were infected with WT *L. pneumophila*, *ΔdotA*, Δ*5* mutant, Δ*5 +plgt2* complement, or Δ5 +plgt2* a strain secreting a catalytically dead Lgt2 effector at an MOI of 150. Cells were then treated for 6 h with thapsigargin (Tg; 1 μg μl^−1^). The level of spliced XBP1 (XBP1s) mRNA was assayed via RT–PCR. (**b**) Three biological replicates of XBP1 mRNA splicing during *L. pneumophila* infection and Tg treatment were quantified. (**c**) HEK-293 FCγ cells were transfected with (1.5 μg) DNA in 24-well dishes with each of the five bacterial effectors Lgt1-FLAG, Lgt2-FLAG, Lgt3-FLAG, SidI-FLAG and SidL-FLAG. Twenty-four hours post-transfection cells were left untreated or treated with Tg (1 μg μl^−1^) for 6 h and RT–PCR was performed to assay for XBP1s mRNA. (**d**) Three biological replicates of XBP1 mRNA splicing in cells transfected with one of the five bacterial effectors Lgt1-FLAG, Lgt2-FLAG, Lgt3-FLAG, SidI-FLAG and SidL-FLAG were quantified. (**e**) RAW267.4 cells were infected with WT *L. pneumophila, ΔdotA*, Δ*5* mutant, Δ*5 +plgt2* complement, or Δ5 +plgt2* at an MOI of 150. Cells were then either untreated or treated with Tg (1 μg μl^−1^) for 6 h. Erdj4 mRNA was monitored via qRT–PCR. Values in all graphs are means±s.e.m. *P<0.05; Student’s *t*-test. Full blots of Fig. 3a–d are provided in Supplementary Fig. 5.

First, we investigated the ER protein IRE1. When unfolded proteins accumulate in the ER, IRE1 oligomerizes and activates its cytosolic ribonuclease domain through auto phosphorylation^35^. Activated IRE1 catalyzes the removal of a 26 nucleotide unconventional intron from XBP1 mRNA^36^,^37^. This causes a frame shift in the XBP1 coding sequence resulting in the translation of XBP1s (spliced) mRNA isoform rather than the XBP1u (unspliced) mRNA isoform. XBP1s is an ER stress-responsive transcription factor as opposed to XBP1u, a protein that negatively regulates XBP1s and terminates the ER stress response^38^. To test whether the processing of XBP1 is affected during *L. pneumophila* infection, we infected cells with WT or *ΔdotA L. pneumophila* strains, and treated them with thapsigargin. Total XBP1 mRNA was harvested and RT–PCR was performed to determine the presence of XBP1u and XBP1s mRNA transcripts. Interestingly, the degree of XBP1u mRNA splicing in cells that were chemically stressed using thapsigargin, was significantly reduced when infected with WT *L. pneumophila*. In contrast, the thapsigargin-induced processing of XBP1u mRNA remained unaffected by *ΔdotA L. pneumophila* infection, indicating that an *L. pneumophila* effector protein may be inhibiting the splicing of XBP1u mRNA (Fig. 4a,b).

Given that the *Δ5 L. pneumophila* mutant strain lacking the five glucosyltransferase effectors was shown to inhibit protein translation, we tested whether any of these effectors had an effect on the stability of the XBP1u mRNA or its splicing during ER stress induction. Strikingly, even though the stability of XBP1 mRNA was not affected by the *Δ5 L. pneumophila* strain; unlike WT *L. pneumophila*, it failed to inhibit XBP1 mRNA splicing in the presence of thapsigargin-induced ER stress (Fig. 4a,b). In contrast, complementation of the *Δ5 L. pneumophila* strain with WT Lgt2 restored the ability to block XBP1u mRNA splicing. This rescue was not observed in a *Δ5* strain complemented with a catalytically dead Lgt2 (Lgt2*) suggesting that the glucosyltransferase activity is needed to block the XBP1u mRNA splicing. It is interesting to note that infection with the *Δ5 L. pneumophila* strain alone was not sufficient to induce XBP1u mRNA splicing. Either this means that the level of XBP1s mRNA generated during infection alone is below the threshold of our detection; or it could imply that XBP1s mRNA may accumulate at a later time point (>6 h) of infection. To test whether any of the glucosyltranferase family effectors are sufficient to inhibit XBP1u mRNA splicing, HEK-293 Fcγ cells were transfected with a plasmid encoding the following FLAG-tagged effectors: Lgt1, Lgt2, Lgt3, SidI and SidL. Cells were then treated with thapsigargin and XBP1u mRNA splicing was monitored. As expected, mock-transfected cells were able to process XBP1u mRNA to its spliced form XBP1s in the presence of thapsigargin. Remarkably, cells transfected with Lgt1, Lgt2 or Lgt3 and treated with thapsigargin showed a defect in the ability to process XBP1u mRNA as evidenced by the absence of the XBP1s form (Fig. 4c,d). SidI and SidL showed little to no effect in blocking XBP1u mRNA splicing. The most significant block in XBP1u mRNA splicing was observed with Lgt1 and Lgt2. This data highlights that Lgt2 and Lgt1 are not only necessary, but also sufficient for the suppression of XBP1u mRNA splicing during ER stress. To ensure that splicing of XBP1u mRNA was indeed inhibited during infection, the mRNA of its downstream target, Erdj4 was assessed via qRT–PCR (Fig. 4e). Interestingly, while uninfected cells showed a twofold induction of Erdj4 mRNA levels after thapsigargin treatment, the cells infected with WT *L. pneumophila* inhibited the induction of Erdj4, suggesting that splicing of XBP1u mRNA has indeed been blocked. As expected, Erdj4 was induced in a *Δ5 L. pneumophila* strain treated with thapsigargin, but was inhibited in a *Δ5 L. pneumophila* strain complemented with WT Lgt2 (Fig. 4e). To rule out an indirect effect of XBP1u mRNA splicing on BiP and CHOP transcripts, we also investigated BiP and CHOP mRNA abundance in FLAG-tagged Lgt2 transfected cells treated with thapsigargin. We found no difference in BiP and CHOP mRNA levels between these cells and mock-transfected cells treated with thapsigargin (Supplementary Fig. 4a,b) suggesting other *L. pneumophila* effectors might be causing upregulation of BiP/CHOP mRNA.

However, given that the glucosyltransferase effector family of *L. pneumophila* has been shown to repress global protein synthesis, we first aimed at delineating the effects of protein translation inhibition with that of XBP1u mRNA splicing.

### Global protein translation inhibition does not mirror *L. pneumophila* infection

**Figure 5:**
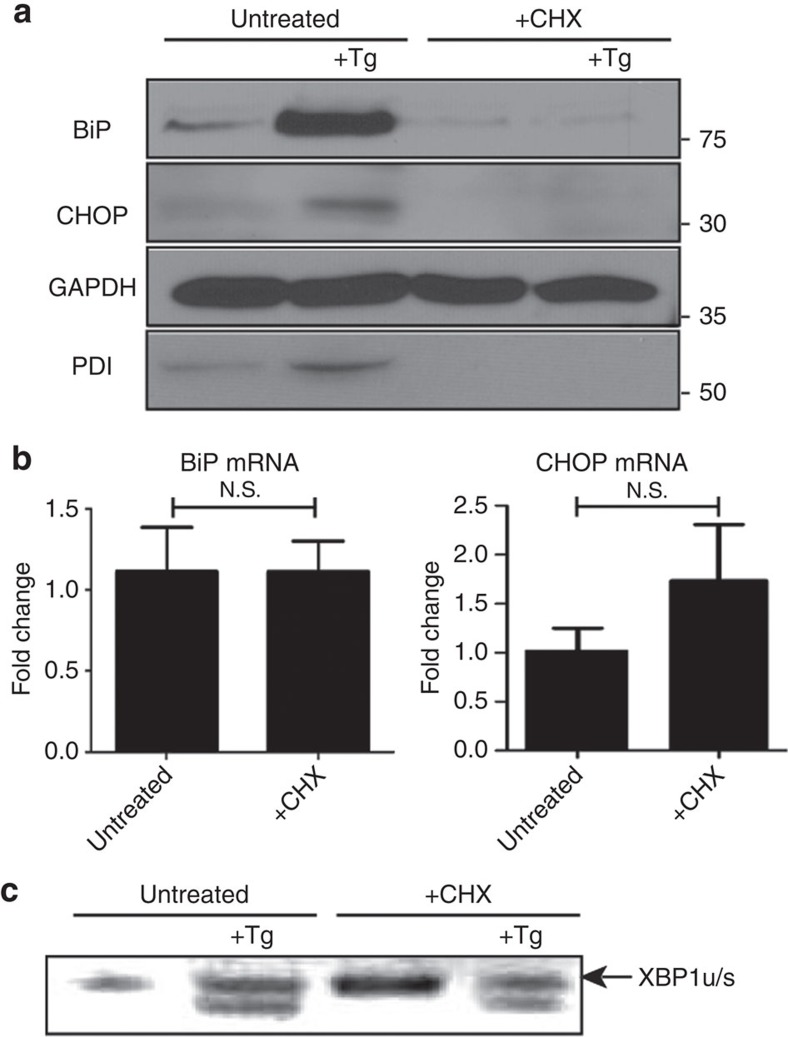
Global protein translation inhibition does not the affect the UPR in the same manner as *L. pneumophila* infection. (**a**) RAW 264.7 cells were left untreated or treated with either thapsigargin (Tg; 1 μg μl^−1^), CHX (1 μg μl^−1^), or a combination of both for 6 h. Protein levels of UPR targets BiP, CHOP and PDI were monitored via immunoblot. (**b**) RAW267.4 macrophage cells were treated with CHX or left untreated and BiP and CHOP mRNA levels were monitored via qRT–PCR. Three internal replicates were performed, values in all graphs are means±s.e.m. *P<0.05; Student’s *t*-test. (**c**) HEK-293 FCγ cells were treated with CHX (1 μg μl^−1^), Tg (1 μg μl^−1^), Tg (1 μg μl^−1^) and CHX (1 μg μl^−1^), or left untreated for 6 h. XBP1u mRNA splicing was monitored via RT–PCR. Full blots for Fig. 5a,b are provided in Supplementary Fig. 5.

In light of *L. pneumophila*’s known ability to target host protein translation, we also assayed whether a chemical inhibitor of protein translation could recapitulate our observations of specific modulation of UPR targets. RAW267.4 cells were treated with cycloheximide (CHX) to inhibit global protein translation; the cells were subsequently treated with thapsigargin to induce ER stress. In contrast to *L. pneumophila* infection, where only the translation of BiP and CHOP was blocked and PDI levels were not affected, CHX treatment led to complete protein translation inhibition and the levels of PDI, CHOP and BiP all decreased similarly (Fig. 5a). Interestingly, CHX treatment alone did not induce the UPR as transcript levels of BiP and CHOP mRNA remain unchanged between untreated and CHX treated cells (Fig. 5b). This observation contrasts WT *L. pneumophila* infection in that infection alone was enough to induce transcription of BiP and CHOP. Next, we investigated whether protein translation inhibition could result in similar block of XBP1u mRNA splicing as was seen with Lgt1 and Lgt2. RAW267.4 cells were treated with CHX and thapsigargin and XBP1u mRNA splicing was monitored. Our data indicate that unlike Lgt1 and Lgt2, CHX treatment in the presence of thapsigargin was not able to block the splicing of XBP1u mRNA (Fig. 5c). This data suggests that block in protein translation alone is not sufficient to inhibit XBP1u mRNA splicing. These data support the idea that although the glucosyltransferase activity of the effectors is necessary for the inhibition of XBP1u mRNA splicing during *L. pneumophila* infection, other *L. pneumophila* effectors might also be inhibiting the UPR by targeting the remaining sensors of UPR (ATF6 and PERK).

### ATF6 is processed during *L. pneumophila* infection

**Figure 6:**
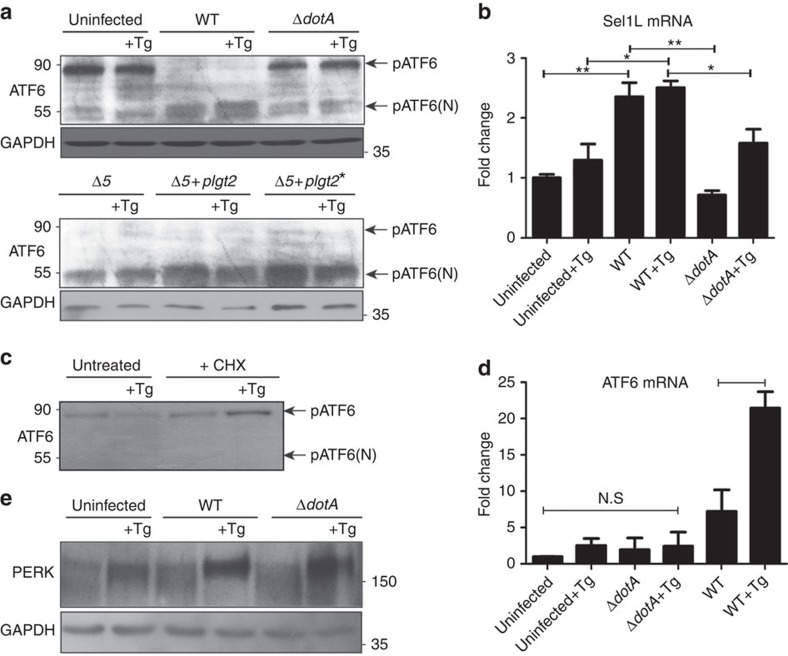
ATF6 is processed during *L. pneumophila*infection. (**a**) RAW 264.7 cells were left uninfected or infected with either WT *L. pneumophila*, *ΔdotA*, B. Δ5, Δ5+plgt2, or Δ5 plgt2* (catalytically dead lgt2) at an MOI of 150. Cells were then left untreated or treated with thapsigargin (Tg; 1 μg μl^−1^) for 6 h. Full-length ATF6 (pATF6) and cleaved pATF6(N) were monitored via immunoblot. Full blots for Fig. 6a are provided in Supplementary Fig. 5. (**b**) HEK-293 FCγ cells infected with either WT *L. pneumophila* or *ΔdotA* and then treated for 6 h with Tg (1 μg μl^−1^). Sel1L mRNA levels were then monitored via qRT–PCR, with GAPDH serving as an endogenous control. Two biological replicates were used each with three technical replicates using the identical experimental conditions. Values in all graphs are means±s.e.m. *P<0.05; Student’s *t*-test. (**c**) Cleaved and uncleaved ATF6 levels were similarly monitored in cells treated with CHX; 1 μg μl^−1^), Tg (1 μg μl^−1^), Tg and CHX, or left untreated for 6 h. Full blots for Fig. 6b are provided in Supplementary Fig. 5. (**d**) RAW267.4 cells were infected with either WT *L. pneumophila* or the *ΔdotA* strain at an MOI of 150 and cells were then treated with Tg (1 μg μl^−1^) or left untreated for 6 h. ATF6 mRNA transcript levels were monitored via qRT–PCR. Two biological replicates were used each with three technical replicates using the identical experimental conditions. Values in all graphs are means±s.e.m. *P<0.05; Student’s *t*-test. (**e**) RAW267.4 cells were infected with either WT *L. pneumophila* or the *ΔdotA* strain at an MOI of 150 and cells were then treated with Tg (1 μg μl^−1^) or left untreated for 6 h. PERK phosphorylation was monitored via immunoblot. GDH served as loading control. Full blots for Fig. 6d are provided in Supplementary Fig. 5.

To further understand the relationship between ER stress and *L. pneumophila* infection, we tested the two additional sensors of the ER: ATF6 and PERK. When misfolded proteins accumulate in the ER, ATF6 translocates to the Golgi, where it is proteolytically cleaved^39^. The cytosolic portion of ATF6 acts as transcription factor that induces the transcription of several UPR genes including BiP/CHOP^40^. Remarkably, we observed that ATF6 was processed more robustly from its uncleaved pATF6 form to its cleaved form pATF6(N) in cells infected with WT *L. pneumophila* compared with those treated with thapsigargin (Fig. 6a). We did not observe any ATF6 processing in uninfected cells, presumably because 6 h of thapsigargin treatment was not sufficient to induce enough ATF6(N) accumulation. It is also known that ATF6 mRNA is upregulated upon thapsigargin treatment which leads to the re-synthesis of full-length ATF6 (ref. 41). In contrast, *L. pneumophila* infection caused very efficient ATF6 processing that resulted in the accumulation of cleaved ATF6(N). Furthermore, when cells were infected with the *Δ5* strain of *L. pneumophila*, ATF6 processing resembled that of WT *L. pneumophila* (Fig. 6a) suggesting that loss of protein translation was not responsible for processing of ATF6. In agreement with this conclusion, when we treated cells with CHX and thapsigargin and monitored the levels of ATF6, we observed that ATF6 was not processed (Fig. 6c). To assess the activity of pATF6(N), we monitored the mRNA levels of Sel1L, a transcriptional target of pATF6(N)^42^. Indeed, we observed that the Sel1L mRNA is significantly upregulated by WT *L. pneumophila* alone (Fig. 6b). This data indicates that the loss of full-length ATF6 during *L. pneumophila* infection is not due to absence of protein turnover but rather cleavage of ATF6 to its active pATF6(N) form.

Previous studies have shown that ATF6 processing is tightly regulated by its association with BiP^43^,^44^. Loss of BiP causes constitutive processing of ATF6 which is congruent with our data in that *L. pneumophila* blocks BiP upregulation and we observe robust ATF6 processing in the presence of *L. pneumophila*. Additionally, this data partially explains why we observed the transcriptional upregulation of both BiP and CHOP mRNA during *L. pneumophila* infection as both are transcriptional targets of processed pATF6(N). To further understand the role of ATF6 during infection with *L. pneumophila* we performed qRT–PCR to determine ATF6 transcript abundance. Interestingly, as observed by the immunoblots of ATF6, WT *L. pneumophila* infection alone was enough to induce a transcriptional upregulation of ATF6. In contrast, *ΔdotA L. pneumophila* showed little or no effect on ATF6. Furthermore, the cellular response to WT *L. pneumophila* was more robust than the uninfected control cells treated with thapsigargin (Fig. 6d).

Finally, we tested the UPR sensor, PERK. During ER stress, PERK activates itself by oligomerization and autophosphorylates its cytosolic domain. This activated cytosolic domain blocks protein translation by directly phosphorylating the α subunit of the eukaryotic initiation factor 2^5^. Our results show that PERK activation occurred normally in the presence of either WT or *ΔdotA L. pneumophila* strains when cells were treated with thapsigargin (Fig. 6e). However, unlike ATF6, *L. pneumophila* infection alone did not lead to an activation of PERK suggesting that *L. pneumophila* was neither upregulating nor inhibiting PERK activity.

## Discussion

Pathogenic intracellular bacteria often utilize an arsenal of effectors to create a host environment that is conducive for their replication and propagation of infection. Many of these pathogens utilize membrane vesicles derived from the endocytic or phagocytic compartments to form these unique intracellular niches. Additionally, modulation of intracellular trafficking in order to recruit ER-derived vesicles is a key strategy for pathogens such as *L. pneumophila*^45^ and *Brucella abortus*^46^.

The host UPR is a critical component of protein homeostasis. The UPR is responsible for responding to ER stress and initiating three general outcomes: reduction in global protein synthesis, upregulation of specific target genes, and in extreme cases, the induction of apoptosis^47^. However, the UPR pathway is not just activated by the accumulation of unfolded proteins. The host can turn on the UPR as an anticipatory stress response to many bacterial and viral infections well before the disruption of protein homeostasis^48^. Given that extreme ER stress leads to apoptosis, it is logical to speculate that it may be advantageous for intracellular pathogens to manipulate or dampen this process in order to gain time for replication. For some pathogens, such as *Vibrio cholerae*, modulation of the UPR has also been shown to be important in controlling the host immune response, although direct manipulation of the UPR has not been characterized^21^. Interestingly, *B. abortus* and *Brucella melitensis*, two intracellular bacterial pathogens that reside in an ER-like organelle, were both shown to activate the UPR^13^,^15^. Despite the close interplay between *Legionella* and the ER, the role of the UPR during *L. pneumophila* infection was poorly understood. Thus, we set out to investigate whether *L. pneumophila* is capable of modulating the host UPR.

Our study shows that the glucosyltransferase family effectors, Lgt1 and Lgt2 have the remarkable ability to target the IRE1 arm of the UPR pathway. More specifically, we discovered that Lgt1 and Lgt2 inhibit the splicing of the XBP1u mRNA transcript which is normally spliced via the endonuclease domain of IRE1. This observation reflects *Legionella*’s strategy to utilize the ER without inducing UPR driven apoptosis. When investigating the activity of the host UPR, we also noted that pATF6, a key sensor of ER stress is processed during *L. pneumophila* infection. pATF6 was cleaved to generate a fragment, pATF6(N), a well-established transcription factor that upregulates UPR elements BiP and CHOP. Indeed, *L. pneumophila* infection alone led to the upregulation of BiP and CHOP transcripts. It is known that dissociation of BiP from ATF6, leads to its translocation into the Golgi where it gets processed to generate the transcription factor pATF6(N). Given that *L. pneumophila* recruits BiP to the LCV and dissociation of BiP from the ER sensors ATF6 and IRE1 leads to their activation, it is possible that the host cells detect the dissociation of BiP as a form of ER stress and activate ATF6 (refs 4, 49). However, *L. pneumophila* is able to suppress the response by inhibiting BiP/CHOP at the level of translation. When we tested whether Lgt2 also had a role in blocking BiP and CHOP expression, the Δ*5* mutant showed no defect compared with WT *L. pneumophila* and this processing was not dependent on the glucosyltransferase effectors Lgt1 and Lgt2, implying that additional effectors are responsible for this effect. This finding is consistent with earlier reports that show that *L. pneumophila* utilizes multiple effectors to target the same cellular pathway^50^. For example, *L. pneumophila* has been shown to modulate host Rab1 GTPase function through at least six effectors^51^,^52^. Furthermore, a chromosomal deletion strain of *L. pneumophila* that lacked 30% of its total genome sequence was still able to survive intracellularly indicating that many *L. pneumophila* effectors share overlapping functions and often target the same pathway^53^.

**Figure 7:**
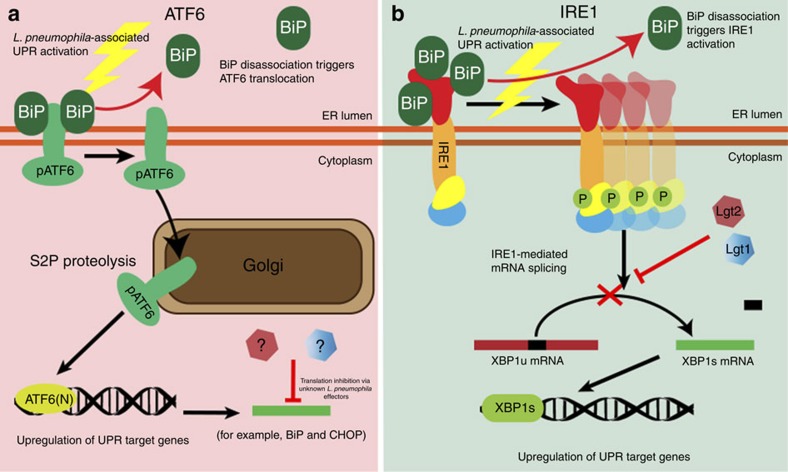
*Legionella* specifically blocks the ATF6 arm and IRE1 arms of the UPR. (**a**) ATF6 is processed from the inactive form pATF6 to ATF6(N) via SP2-mediated proteolysis at the Golgi, in the presence of WT *L. pneumophila* and this leads to the upregulation of unfolded protein response genes BiP and CHOP. Currently unknown *L. pneumophila* effectors can block the translation of these genes to inhibit the UPR. (**b**) The *L. pneumophila* effectors Lgt1 and Lgt2 are necessary and sufficient to block the UPR endonuclease protein IRE1-mediated XBP1 mRNA splicing during infection. Under normal conditions XBP1 is spliced to the XBP1s form with is translated to the XBP1 transcription factor that targets UPR related genes.

Taken together, our data shows that host cells detect *L. pneumophila* infection as a form of ER stress and attempt to upregulate the UPR target genes BiP and CHOP, presumably to aid in protein folding and the eventual induction of apoptosis. However, *Legionella* utilizes multiple effectors to suppress the induction of the UPR. In this study, we have identified the first bacterial effectors (Lgt1 and 2) that are sufficient to block XBP1u mRNA splicing without affecting the remaining branches of the UPR, ATF6 and PERK. A strain lacking both these effectors (Δ*5* mutant) was unable to block XBP1u mRNA splicing, suggesting that they are necessary for *L. pneumophila* to mediate IRE1 inhibition during infection. These data are summarized in our model (Fig. 7). Currently, we are focused on identifying additional effectors that block the expression of BiP and CHOP as well as understanding how Lgt2 and Lgt1 block IRE1-mediated XBP1u mRNA splicing. Given the close interplay between IRE1 and the inflammatory response pathway, it is possible that TLR signalling may play a role in the upregulation of UPR related genes^48^. Thus, it would be exciting to further investigate how *L. pneumophila* uses the UPR to modulate the host innate immune system. In conclusion, we believe that the data presented in this paper are critical in understanding the intimate relationship of *Legionella* with the host ER membrane and will open a new avenue of research that can utilize Lgt1 and Lgt2 as effective tools to study XBP1u mRNA splicing during ER stress.

## Methods

### Bacterial strains

All *Legionella* and *Escherichia coli* strains were gifts from Craig Roy’s laboratory at Yale University. *Legionella* strains used in this study were routinely cultivated on Charcoal Yeast Extract (CYE) agar. The antibiotic Streptomycin was used at a concentration of 100 μg ml^−1^ when needed. Lgt1, Lgt2, Lgt3, SidI and SidL were cloned from genomic DNA prepared from WT *L. pneumophila* (Lp01) into pcDNAT/40 3XFlag Vector, a gift from Craig Roy’s laboratory. Plasmids were recovered from the *E. coli* strain MAC1.

### Cell culture

All mammalian cell lines were obtained from Craig Roy’s laboratory at Yale University. HEK-293 FCγ cells were cultured in DMEM (Life Technologies) supplemented with 10% fetal bovine serum (FBS) at 37 °C and 5% CO_2_. RAW 264.7 macrophages were cultured in Roswell Park Memorial Institute media (RPMI) (Corning) supplemented with 10% FBS at 37 °C and 5% CO_2_. Caspase 1/11 (−/−) primary bone marrow derived macrophage cells were a gift from Anita Sil’s laboratory at UCSF, differentiated and frozen in liquid nitrogen and were cultured in media with the following final concentrations: 20% FBS, 10% CMG (conditioned medium supernatant obtained from CMG cells which are NIH/3T3 fibroblasts transfected with recombinant M-CSF), 1.6 mM glutamine, 1 mM Na–Pyruvate and.2 M Penicillin–Streptomycin in DMEM media without phenol red. Cultures of *Legionella* were prepared in DMEM at a multiplicity of infection of 150 bacteria per cell. Bacteria and cells were centrifuged at 500*g* for 5 min at room temperature and then incubated for 1 h at 37 °C and 5% CO_2_. Cells were washed and fresh medium replaced. Rapamycin was used at (10 nM) final concentration where indicated.

### Quantitative RT–PCR

HEK-293 FCγ cell mRNA was harvested using Trizol according to the Life Technologies’ protocol after 6 h of infection. RNA samples were treated with DNAase before reverse transcription with Omniscript (Qiagen). cDNA reactions were primed with Sigma Aldrich poly 20dT. Relative Quantitative PCR was performed using Syber Green. Endogenous GAPDH mRNA was used for normalization. Uninfected and untreated HEK-293 FCγ cells were used as the endogenous control for each qPCR analysis. The following RT–qPCR primers were used: BiP (Mouse) forward-ACTTGGGGACCACCTATTCCT, reverse- ATCGCCAATCAGACGCTCC; BiP (Human) forward-CATCACGCCGTCCTATGTCG, reverse-CGTCAAAGACCGTGTTCTCG; CHOP (Mouse) forward-CTGGAAGCCTGGTATGAGGAT, reverse-CAGGGTCAAGAGGTAGTGAAGGT; CHOP (Human) forward-GGAAACAGAGTGGTCATTCCC, reverse-CTGCTTGAGCCGTTCATTCTC; P115 (Human) forward-GCTTTGTGACAGAGTAGCTTCA, reverse-CCACTTCCAAGCGGTATTTCT; ATF6 (Human) forward-TCCTCGGTCAGTGGACTCTTA, reverse-CTTGGGCTGAATTGAAGGTTTTG; Mouse Erdj4 forward-CTCCACAGTCAGTTTTCGTCTT, reverse-GGCCTTTTTGATTTGTCGCTC.

### Western blot analysis

Infected mammalian cells were lysed in radioimmunoprecipitation assay buffer with the addition of protease (Roche cOmplete), and phosphatase inhibitors (GB Sciences). Protein levels of lysates were determined using the BioRad DC/RC assay. Equal amounts of protein lysate were boiled with SDS load buffer. Equal amounts of protein were loaded and immunoblotting with, Thermo GAPDH antibody was used as a loading control. Antibodies used in assay are as follows: CHOP (Thermo MA1-250; 1:2,000), BiP (Protein Tech Group 11587-1-AP; 1:5,000), Phospho-Perk (Thermo MA5-15033; 1:1,000), GAPDH (Thermo MA5-15738; 1:10,000), PDI (Enzo ADI-SPA-891; 1:5,000), P115 (Lab generated; 1:2,500), p-AKT Serine 473 (Cell Signaling 9271S; 1:1,000).

### Infection

Macrophages were plated at a density of 1 × 10^7^ cells per 100 mm dish. Cells were infected at an multiplicity of infection (MOI) of 150. If cells required opsonization, a lab-generated anti *L. pneumophila* antibody was used at 1:2,000 for 20 min incubation. Immediately after infection, cells were centrifuged for 15 min at 400*g*. After centrifugation, cells were left at 37 °C for an additional 45 min. After a total elapsed time of 1 h cells were washed three times with PBS. RMPI supplemented with 10% FBS or the same media supplemented with thapsigargin (1 μg μl^−1^) or cyclohexamide (1 μg μl^−1^) was replaced after the washes in PBS. Dithiothreitol and Tunicamycin were used at 1 mM and 1 ug μl^−1^ respectively.

### Statistical analysis

Prism software was used for statistical analysis. Where statistical analysis was performed an unpaired student’s *t*-test was performed using three biological replicates. Statistical significance (*) was determined as a *P*-value<0.05.

### Replicative vacuole assay

RAW cells were infected at an MOI of 25 using 24-well dishes with poly-lysine coverslips with cells seeded to a density of about 150,000 per well. Cells were fixed at 1 h post infection and were also fixed at 10 h post infection. Rifampicin (2 μg μl^−1^) was used in cells to clear infections where indicated. Cells were fixed in 4% paraformaldehyde (PFA). A polyclonal rabbit antibody generated against *L. pneumophila* (a gift from Craig Roy’s laboratory) was used for detection of *L. pneumophila* and Hoechst was used to stain nuclei.

### XBP1 RT–PCR analysis

Total mRNA was harvested from HEK-293 FCγ cells. Reverse transcription was performed via Omniscript Reverse Transcriptase. To assess the abundance of XBP1u and XBP1s mRNA transcripts iProof polymerase was used using the following primers: XBP1 forward: ACACGCTTGGGGATGAATGC and XBP1 reverse: CCATGGGAAGATGTTCTGGG. Of special note is that final extensions should be avoided and for visualization a 3% agarose gel consisting of half low melting point agarose is required (Supplementary Fig. 6). Thermo cycle conditions were as followed: (1) 95 °C per 2 min, (2) 95 °C per 20 s, (3) 60.5 °C per 30 s, (4) cycle to step 2, 28 times, (5) 72 °C per 30 s, (6) 4 °C per infinite.

## Additional information

**How to cite this article:** Treacy-Abarca, S. & Mukherjee, S. *Legionella* suppresses the host unfolded protein response via multiple mechanisms. *Nat. Commun.* 6:7887 doi: 10.1038/ncomms8887 (2015).

## Supplementary Material

Supplementary Information Supplementary Figures 1-6
