# Supplementary material for: Legionella suppresses the host unfolded protein response via multiple mechanisms
Source: Nat Commun. 2015 Jul 29;6:7887. doi: 10.1038/ncomms8887 (PMC4519984; doi:10.1038/ncomms8887)
Supplement: Supplementary Information — Supplementary Figures 1-6 [file ncomms8887-s1.pdf]

Supplemental Figure 1:

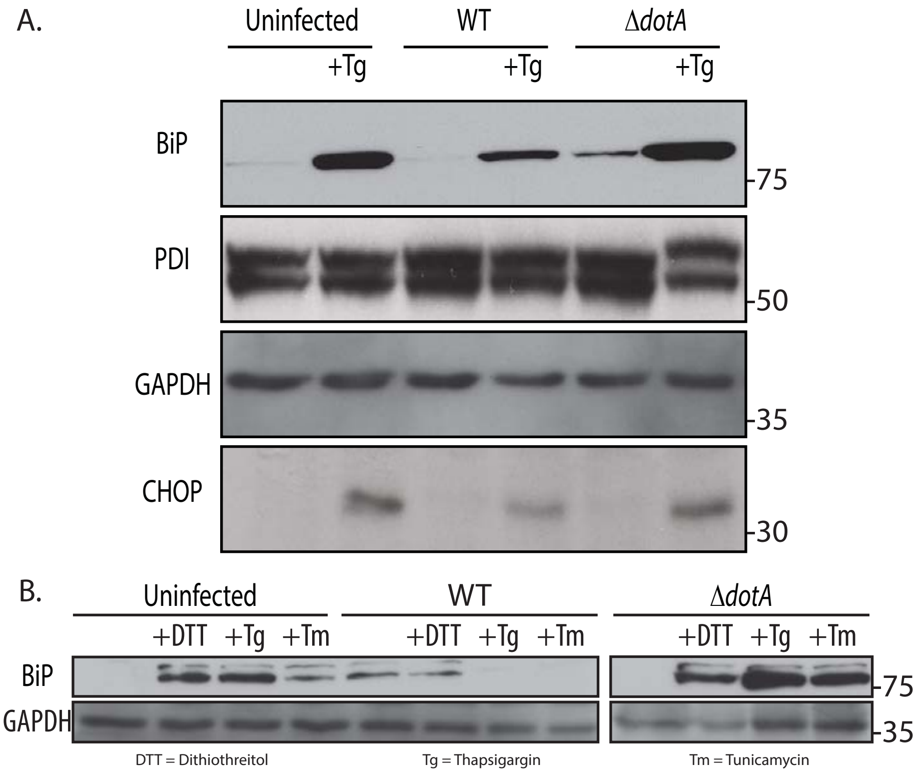

Supplemental Figure 1: The expression of BiP and CHOP is suppressed by *L.p.*

(A) BiP, PDI, and CHOP levels were assayed via immunoblot from RAW 264.7 cells left uninfected or infected with *WT Legionella* (*L.p.*) or Δ*dotA L.p*. at an MOI of 150 while in the presence or absence of Thapsigargin (Tg) (1ug/ul). B. HEK-Fcγ cells were left uninfected or infected with the indicated strain of *L.p.* at an MOI of 150. Subsets of these cells were then treated with DTT (1mM), Tunicamycin (1ug/ul), or Tg for 6 hours and BiP levels were accessed via immunoblot. Full blots are provided in Supplemental Figure 6 for all images.

Supplemental Figure 2:

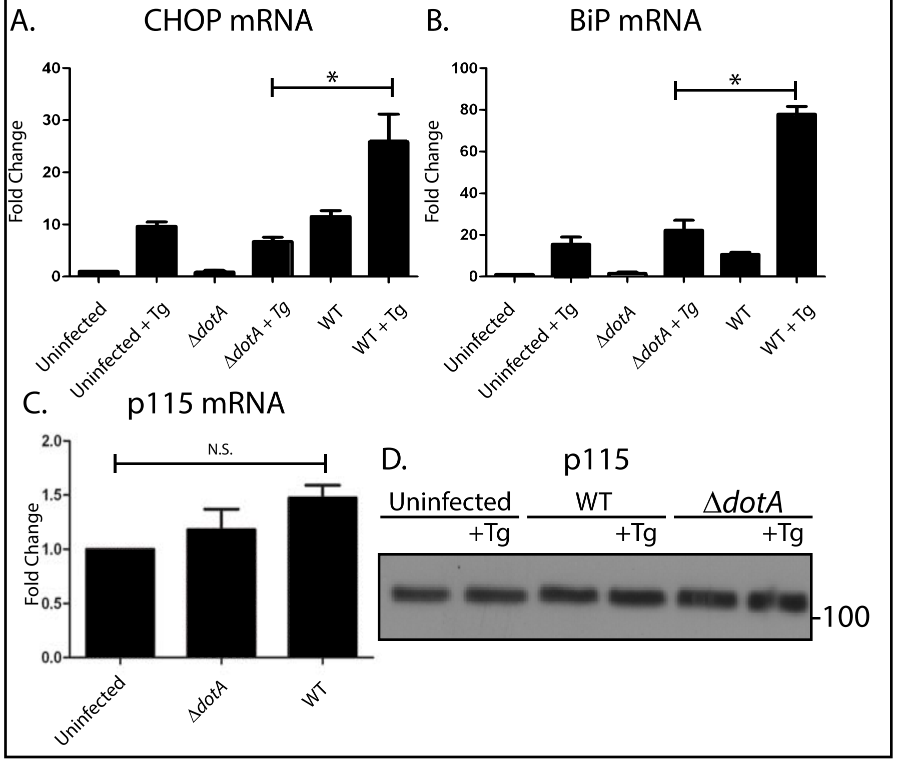

Supplemental Figure 2: The transcription of BiP and CHOP is up-regulated during *L.p.* infection

(A-B) BiP and CHOP mRNA was quantified in HEK-293Fcγ cells infected with either *WT L.p*., ΔdotA L.p., at an MOI of 150 or were left uninfected. Thapsigargin (Tg) (1ug/ul) was applied for 6 hours. qRT-PCR was used to assay for specific mRNA abundance and GAPDH was used as the endogenous control. Three biological replicates with three internal technical replicates were used. Values in all graphs are means ± s.e.m. *$P<0.05$; Student's t-test. (C) The mRNA level of the Golgi protein p115 was also quantified using qRT-PCR from uninfected HEK-Fcγ cells, or cells infected with either WT L.p. or Δ*dotA L.p*. at an MOI of 150. GAPDH was used as an endogenous control. Two biological replicates each with three internal technical replicates were used. D. P115 protein levels were assayed via immunoblot in HEK-293 cells infected with either *WT L.p.* or Δ*dotA,* at an M.O.I. of 150. Subsets of these cells were treated with Tg (1ug/ul). Full blots are provided in Supplemental Figure 6 for all images.

Supplemental Figure 3:

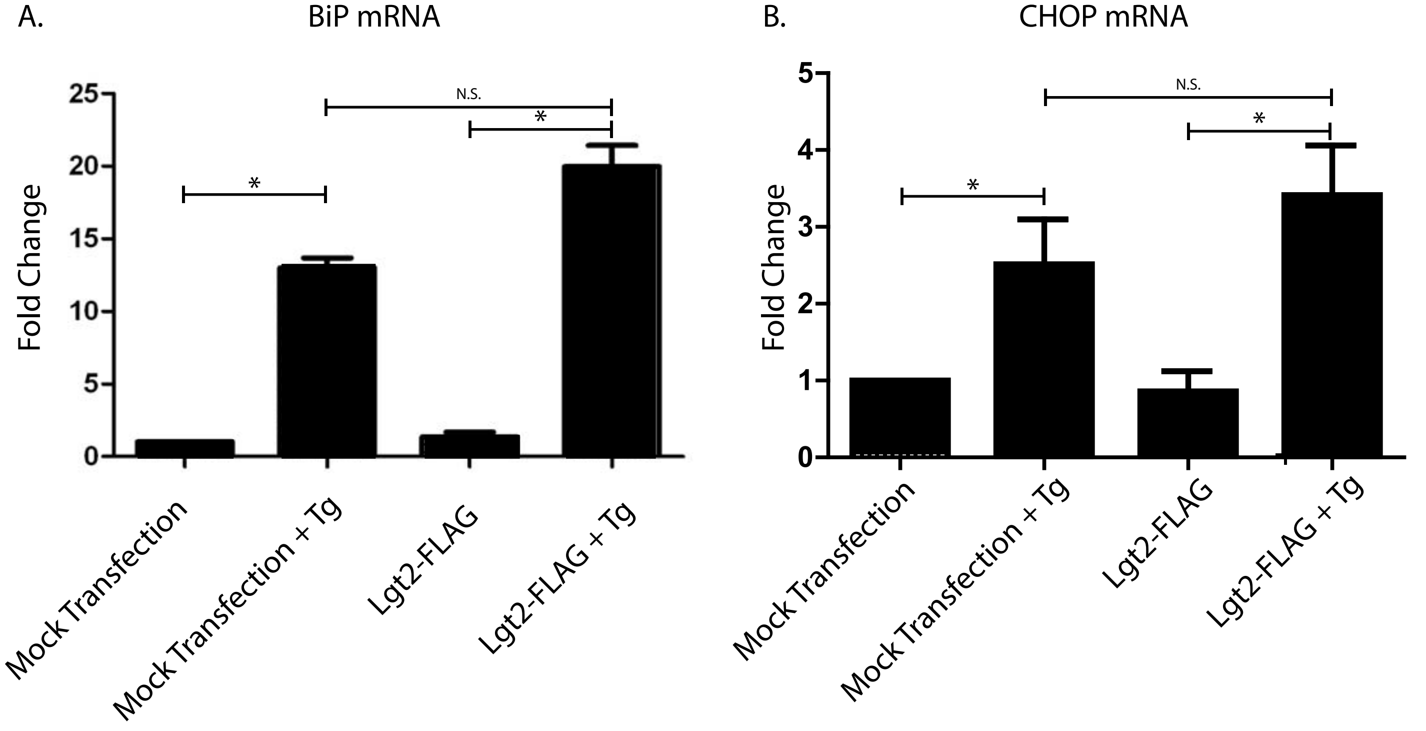

Supplemental Figure 3: Lgt2 does not affect mRNA stability of BiP and CHOP

(A-B) BiP or CHOP mRNA were monitored via qRT-PCR during transfection of HEK-Fcγ cells with the *L.p.* effector construct Lgt2-FLAG. Tg used at (1ug/ul). GAPDH was used as a endogenous control. Data are derived from two biological replicates, each with three internal technical replicates. Values in all graphs are means ± s.e.m. *$P<0.05$; Student's t-test.

Supplemental Figure 4:

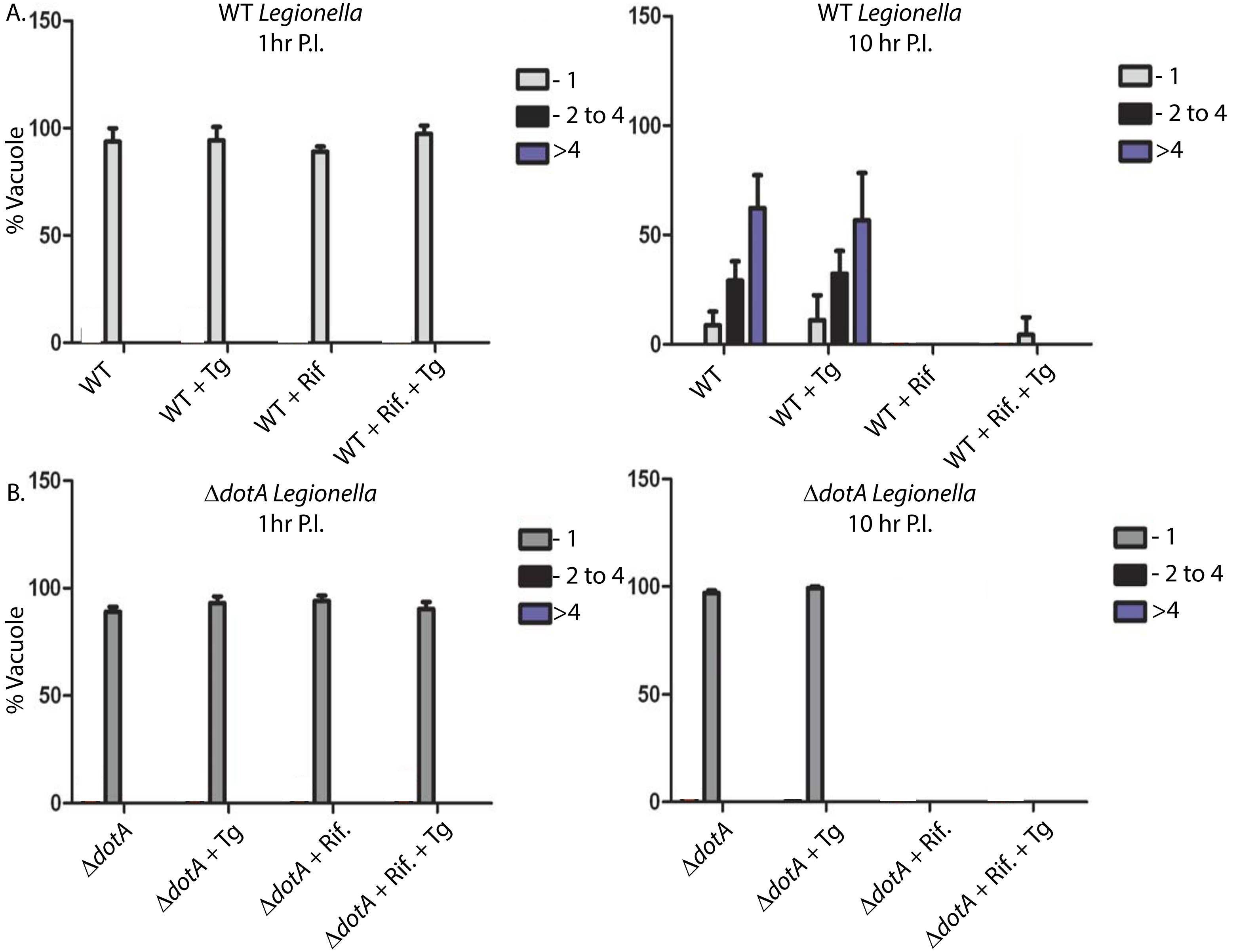

Supplemental Figure 4: Rifampicin treatment effectively clears *Legionella*

(A) Raw 264.7 cells were infected with A. *WT L.p*. or B. Δ*dotA L.p*. at an MOI of 25. Cells were then treated with Tg (1ug/ul), Rifampicin (Rif) (2ug/ul), or Tg and Rif. After 11 hours of infection cells were fixed in 4% PFA. Intracellular replication was assayed by counting the number of *L.p.* within a replicate vacuole. The data is presented using bins, 1, 2 to 4, or >4 L.p. The data was gathered from three replicates which were pooled. Values in all graphs are means ± s.e.m..

Supplementary Figure 5:

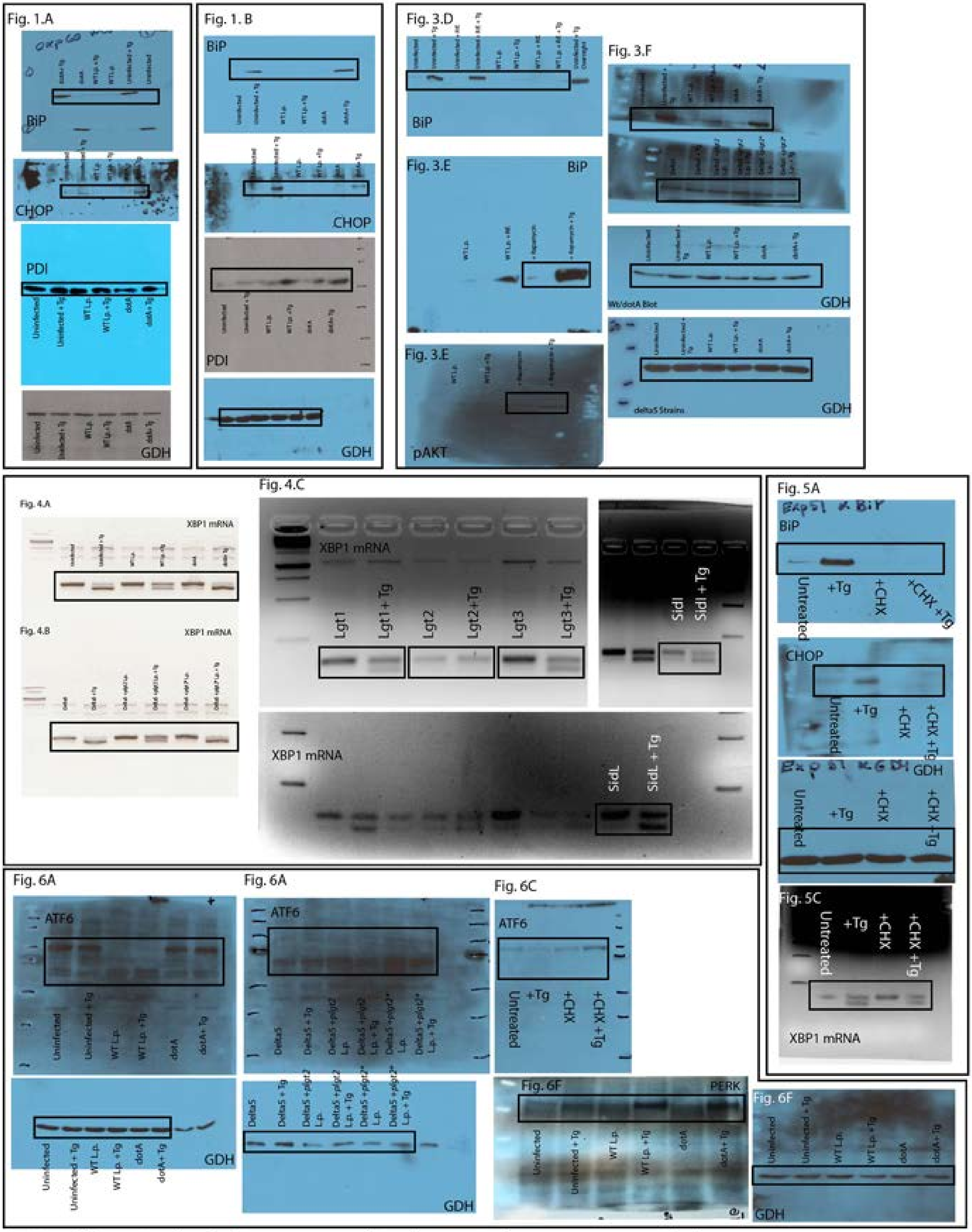

Supplemental Figure 5: Original images used throughout the main figures

All images used in the main figures in their original sizes.

Supplental Figure 6:

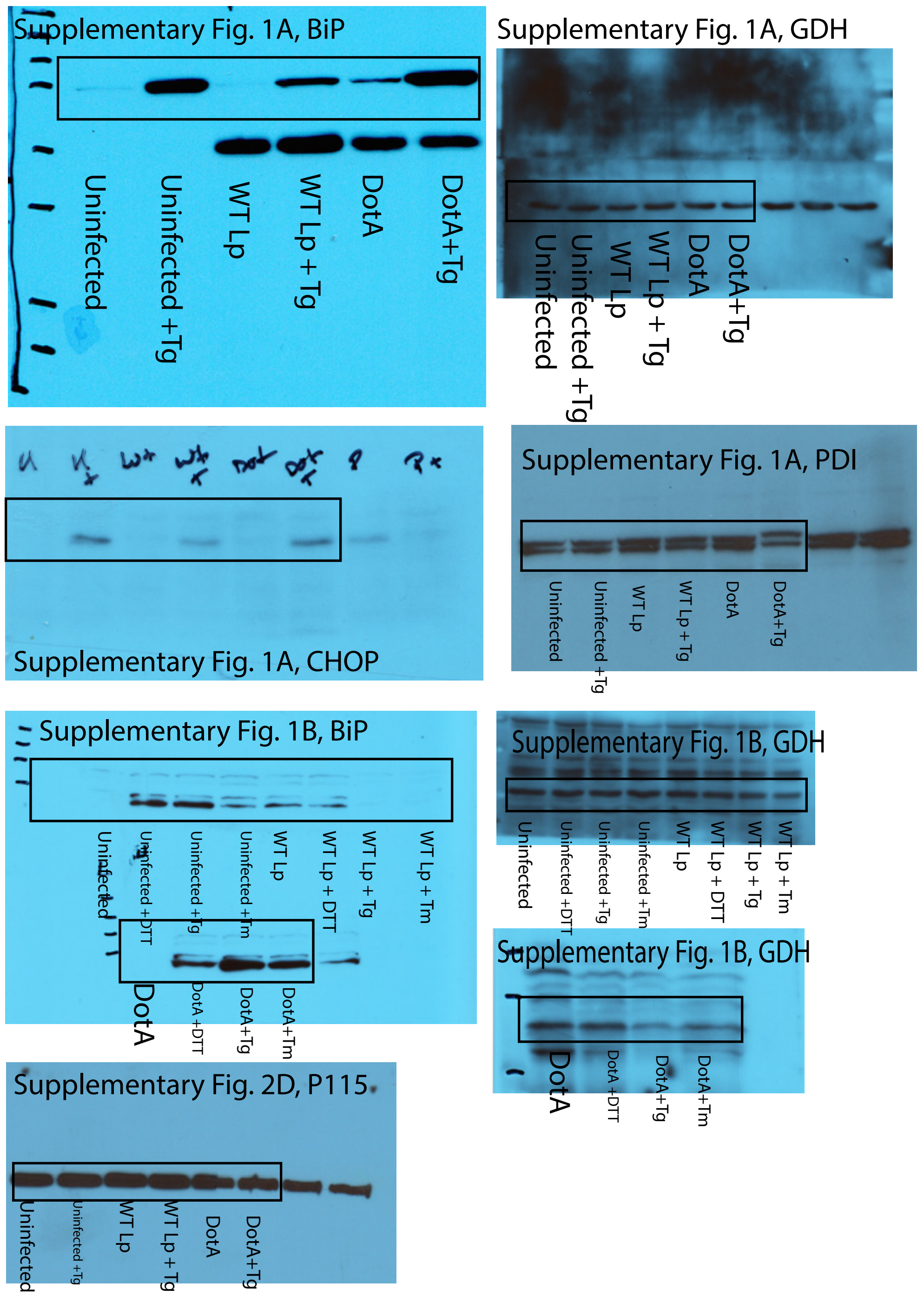

Supplemental Figure 6: Original images used throughout the supplementary figures

All images used in the supplemental figures in their original sizes.
